# Association of telomere length and mitochondrial DNA copy number with risperidone treatment response in first-episode antipsychotic-naïve schizophrenia

**DOI:** 10.1038/srep18553

**Published:** 2015-12-18

**Authors:** Zongchang Li, Maolin Hu, Xiaofen Zong, Ying He, Dong Wang, Lulin Dai, Min Dong, Jun Zhou, Hongbao Cao, Luxian Lv, Xiaogang Chen, Jinsong Tang

**Affiliations:** 1 Mental Health Institute of the Second Xiangya Hospital, Key Laboratory of Psychiatry and Mental Health of Hunan Province, Central South University, Hunan, Changsha 410011, China; 2 The State Key Laboratory of Medical Genetics, Central South University, Changsha, Hunan, China; 3 Unit on Statistical Genomics, National Institute of Mental Health, NIH, Bethesda 20892, USA; 4 The National Clinical Research Center for Psychiatric and Psychological Diseases, Changsha, China; 5 Department of Psychiatry, The Second Affiliated Hospital of Xinxiang Medical University, Xinxiang, Henan, China

## Abstract

Accumulating evidence indicates a putative association of telomere length and mitochondrial function with antipsychotics response in schizophrenia (SCZ). However, pharmacological findings were limited and no previous work has assessed this in a prospective longitudinal study. This study assessed telomere length and mitochondrial DNA copy number in first-episode antipsychotic-naïve SCZ patients with 8-week risperidone treatment to evaluate the association between these biomarkers and clinical treatment response. We recruited 137 first-episode antipsychotic-naive SCZ patients (and 144 controls) at baseline and 89 patients completed the 8-week follow-up. Patients, completed follow-up, were divided into Responders (N = 46) and Non-Responders (N = 43) according to the percentage of symptoms improvement. Linear regression analyses show that SCZ patients had significantly lower mtDNA copy number (β = −0.108, p = 0.002), and no alteration of telomere length when compared with healthy controls. In addition, compared with Non-Responders, Responders had significantly lower mtDNA copy number (β = −0.178, p = 0.001), and longer telomere length (β = 0.111, p = 0.071) before the 8-week treatment. After treatment, Responders persisted lower mtDNA copy number comparing with No-Responders (partial η^2^ = 0.125, p = 0.001). These findings suggest that telomere length and mtDNA copy number may hold the potential to serve as predictors of antipsychotic response of SCZ patients.

## Introduction

Cellular aging is a state of irreversible cell cycle arrest and actively contribute to degenerative pathologies and somatic senescence. Emerging evidence documents cellular aging associated with some medical and psychiatric conditions including cardiovascular disease, diabetes, neurodegenerative diseases, SCZ and some other psychotic disorders^1^,^2^,^3^,^4^,^5^. Telomere erosion and mitochondrial dysfunction are two well-known molecular pathways implicated in the key process of cellular aging. Telomeres are tandem repeats of the sequence TTAGGG at the end of eukaryotic linear chromosomes; they enable chromosome ends to be distinguished from sticky double-stranded breaks and protect the chromosomes from natural cellular degradation and end-to-end fusion^6^,^7^. In normal somatic cells, telomeres progressively shorten with each cell division because of the replication problem for the ends of linear DNA and the down-regulated activity of telomerase with limited ability to counteract the eroded strand. When the length of telomere reaches critically short, the cell begins its senescence and apoptosis^8^. Thus, telomere length may serve as a biological time recorder to indicate the replicative capacity of a cell along its division.

Mitochondria are key organelles of the eukaryotic cells and carry essential functions in energy metabolism, calcium homeostasis and apoptosis^9^. In addition, mitochondria are also the major intracellular source for generation of reactive oxygen species (ROS) and the primary target of ROS-induced oxidative damage^10^. In mitochondria, mitochondrial DNA (mtDNA) is highly susceptible to oxidative damage due to high levels of cellular ROS, lack of protective histones and limited DNA repair capacity^11^,^12^,^13^. In most cases, the content of mtDNA accurately modulated during physiological processes. However, abnormal variation may occur when the mitochondrial microenvironment was changed. The alteration of mtDNA copy number has been suggested as a sensitive index of cellular oxidative stress, mitochondrial dysfunction, aging process and age-related diseases^14^.

Recent studies have revealed shared pathways in modulating telomere length and mitochondrial biogenesis^15^,^16^. Telomere dysfunction activates p53-mediated pathway to repress the expression of peroxisome proliferator-activated receptor gamma, coactivator 1 alpha and beta (PGC-1α and PGC-1)^15^. The repression of both co-activators impairs functional mitochondrial biogenesis leading to increased levels of ROS damaging both telomere and mitochondrial DNA, and starts a negative feedback loop. In addition, telomerase reverse transcriptase (TERT) serves as a catalytic subunit of telomerase with canonical role of telomere maintenance. TERT contains both nuclear localization signal and mitochondrial targeting sequence, and might be transported from nuclei to mitochondria under increased oxidative stress conditions to regulate mitochondrial function and protect mtDNA from oxidative damage^17^. Moreover, several studies reported lately that positive correlations have been observed between telomere length and mtDNA copy number variations in healthy adults^18^,^19^.

Emerging evidence indicates that physiological changes may relate to premature aging that happens in SCZ, and it is tempting to speculate that SCZ might be a syndrome of accelerated aging^20^. Given the intimate link between telomere-mediated and mitochondria-mediated pathways and the aging processes, it assumes that the alterations of telomere length and mitochondrial DNA copy number might implicate in SCZ. Several studies have evaluated the influence of telomere length and mtDNA copy number on SCZ^21^,^22^,^23^,^24^,^25^,^26^,^27^,^28^,^29^,^30^. However, results are controversial to each other due to the high heterogeneity of SCZ subjects. It has been suggested that antipsychotic treatment may lengthen telomere and affect the function of mitochondrial in SCZ patients. Several cross-sectional studies showed that patients with longer telomere tend to better respond to antipsychotic treatment^25^,^31^. On the other hand, treatment responsive SCZ subjects had fewer mitochondria per synapse in striatum^32^. Therefore, we hypothesize that: (1) accelerated telomere erosion and mtDNA copy number variations may be associated in first-episode antipsychotic-naive SCZ; (2) antipsychotics may have influence on both telomere and mtDNA; (3) the telomere length and mtDNA copy number may be used to predict if a first-episode SCZ patient be antipsychotic-responsive or not.

In this study, we test our hypothesis with a longitudinal design. We measured the telomere length and mtDNA copy number in first-episode antipsychotic-naïve SCZ patients followed by a 8-week risperidone treatment, and examined whether those two parameters were associated with the treatment-resistant character in SCZ patients.

## Results

**Figure 1:**
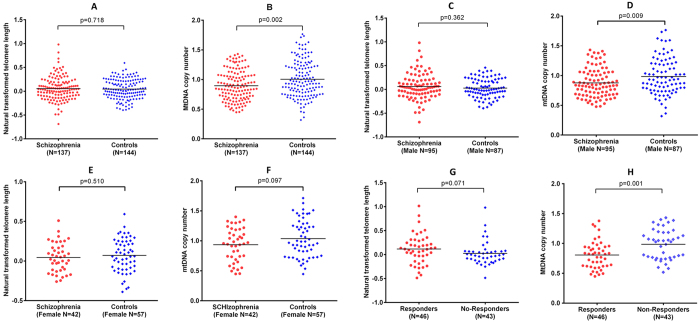
Telomere length and mtDNA copy number in different groups. **(A**) Telomere length in schizophrenia patients and healthy controls. (**B**) MtDNA copy number in schizophrenia patients and healthy controls. **(C**) Telomere length in male subjects in schizophrenia patients and healthy controls. (**D**) MtDNA copy number in male subjects in schizophrenia patients and healthy controls. **(E**) Telomere length in female subjects in schizophrenia patients and healthy controls. (**F**) MtDNA copy number in female subjects in schizophrenia patients and healthy controls. (**G**) Telomere length in Responders and No-Responders. (**H**) MtDNA copy number in Responders and No-Responders. Horizontal bars represent the mean values.

**Figure 2:**
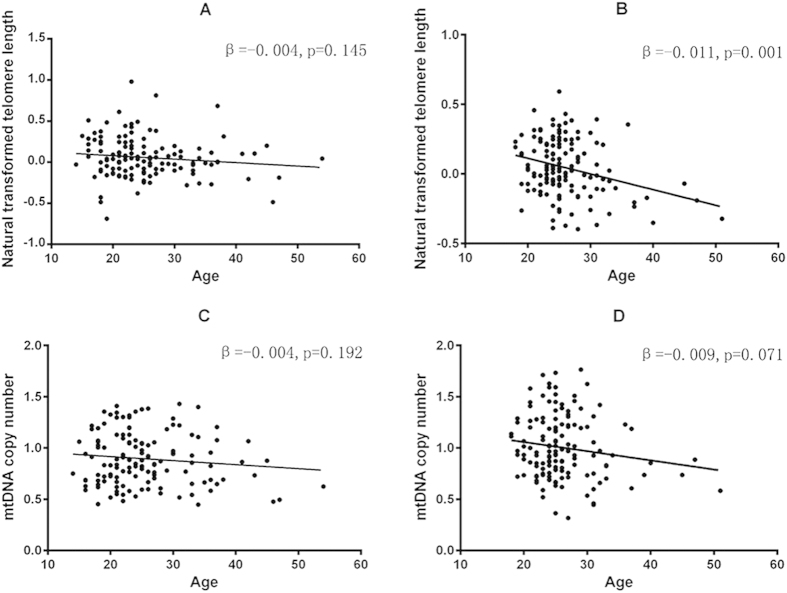
Correlations of age with telomere length or mtDNA copy number in schizophrenia patients and controls at baseline. (**A**) Correlation between baseline telomere length and age in schizophrenia patients. (**B**) Correlation between baseline telomere length and age in controls. (**C**) Correlation between baseline mtDNA copy number and age in schizophrenia patients. (**D**) Correlation between baseline mtDNA copy number and age in controls.

A total of 137 first-episode antipsychotic-naïve SCZ patients and 144 healthy controls were recruited in this study. Table 1 presents the demographic data of SCZ patients at baseline and healthy controls. Comparisons of baseline telomere length and mtDNA copy numbers in SCZ group with that in control group were shown in Fig. 1(A,B). We observed significant lower baseline mtDNA copy numbers in SCZ patients compared with that in health controls (β = −0.108, p = 0.002). However, the telomere length showed no significant difference between the SCZ and control groups (β = 0.010, p = 0.718). Linear regression analysis showed that sex has no significant effect on telomere length and mtDNA copy numbers over all samples (Table 2). However, male SCZ patients demonstrated significant lower mtDNA copy numbers compared with healthy controls, while female patients only showed milder decreased mtDNA copy numbers (Fig. 1D,F). We detected no significant differences in terms of telomere length when compare SCZ patients and controls in sex-stratified analyses (Fig. 1C,E). Age was inversely associated with telomere length and mtDNA copy number (β = −0.007, p = 0.002; β = −0.006, p = 0.031) in all subjects (Table 2). In addition, similar trends were observed in control group, but not in SCZ group (Fig. 2). Pearson correlation analysis found a significant correlation between the mtDNA copy number and telomere length (r = 0.200, p = 0.016) in controls, but not in SCZ patients (r = −0.003, p = 0.972).

At the endpoint of 8 weeks, a clinical follow-up was completed on 89 patients to evaluate clinical improvement, and a subset of 83 patients also repeated the biomarker measurements. Using the above-mentioned diagnostic criteria, 46 patients were classified as Responders and 43 as No-Responders. There were no significant differences in PANSS positive symptoms (25.52 ± 4.92 vs 24.47 ± 4.39, p = 0.289), PANSS negative symptoms (21.35 ± 6.47 vs 19.21 ± 5.98, p = 0.110), and PANSS general symptoms (46.43 ± 8.56 vs 47.16 ± 8.21, p = 0.684) at baseline between Responders and No-Responders.

We found that Responders had significantly lower mtDNA copy numbers (β = −0.178, p = 0.001), and milder longer telomere length (β = 0.111, p = 0.071) when compared with No-Responders (Fig. 1G,H). Repeated measures ANOVAs showed a significant group effect for the mtDNA copy number (partial η^2^ = 0.125, p = 0.001), indicating that Responders, compared with Non-Responders, showed persistent lower mtDNA copy numbers across the 2 time points. In contrast, no significant group effect for the telomere length was observed (partial η^2^ = 0.033, p = 0.101). There was no time effect on mtDNA copy number (partial η^2^ = 0.032, p = 0.107) and telomere length (partial η^2^ = 0.013, p = 0.316), with no group by time interaction (p = 0.466 and p = 0.816, respectively), indicating no significant antipsychotic effects on mtDNA copy number and telomere length in the 2 groups of patients.

We also performed exploratory analyses of the relationship between baseline telomere length and mtDNA copy number, and symptom severity and symptom improvement in patients. We found that symptom severity was not significantly correlated with the mtDNA copy numbers and telomere length in SCZ patients (Table 3). In addition, multiple linear regression analyses showed that clinical improvement was positively correlated with the baseline telomere length (β = 0.194, p = 0.045) and negatively correlated with the baseline mtDNA copy number (β = −0.267, p = 0.007), indicating that longer telomere length and lower mitDNA copy number at baseline are associated with better symptoms improvement in patients.

## Discussion

In this study, our results show markedly lower mtDNA copy numbers in SCZ compared with healthy controls, indicating a potential role for mitochondrial impairment in the pathogenic pathways of SCZ. This finding is bolstered by prior similar studies for significantly decreased mitochondrial number in brain tissues and peripheral lymphocytes^33^,^34^,^35^. However two previous postmortem studies reported no anomalous mtDNA copy number in brain tissues of SCZ^28^,^29^. Several possibilities might explain the above inconsistency. One explanation is that the mtDNA copy number was measured in different tissues cells, which may influence the outcomes. Another possible explanation is that agonal duration as a confounder may conceal the indigenous alteration of mtDNA in postmortem studies^36^. Finally, we cannot exclude the possibility that the discrepancy may be caused by sample size. We tested 281 subjects whereas the previous studies have relatively smaller sample size (30 and 89 subjects, respectively).

Many studies reported short telomere length in SCZ^21^,^25^,^26^,^30^. However, several other studies failed to replicate this finding^22^,^23^,^24^. In our study, we did not find any alteration of telomere length in first-episode antipsychotic-naïve SCZ when compared with healthy subjects. Some confounders including illness duration, age of subjects, lifestyle and antipsychotics use might partly explain these conflicting results. Alternatively, reduced telomere length may not be an intrinsic feature of mental disorders but a result of cumulative exposure to chronic stress^37^,^38^. In this sense, telomere erosion may be mild in early-phase SCZ, since these individuals undergoing short-term exposure to stress. Therefore, we cannot exclude the possibility that the stress-mediated telomere erosion might be too tiny to be detected in our patients because they had much younger age and shorter illness duration than the patients in previous studies.

Consistent with previous studies, our data in control population showed a strong association between telomere length and mtDNA copy number, and further supports a shared pathway in governing telomere and mitochondrial biogenesis in cellar aging^18^,^19^. However, this association was not observed in SCZ group implying that the pathways shared by regulating telomere length and mitochondrial biogenesis might be disturbed by the pathophysiology of SCZ.

The intriguing findings of this study show that telomere length and mtDNA copy number at baseline may predict risperidone treatment response or clinical improvement at the 8 weeks follow-up in first-episode antipsychotic-naive SCZ patients. Although the molecular mechanism linking telomere and mitochondria to schizophrenic symptom is poorly understood, several cross-sectional studies have suggested that telomere length and mitochondria number might be associated with antipsychotic response in SCZ^25^,^32^. Evidence supported that patients with severer initial illness may be better treated by antipsychotics^39^. Therefore, we hypothesized that patients with longer telomere or lower mtDNA copy number may have severer schizophrenic symptom, and this may indicate better clinical improvement. However, this interpretation is not supported by our data. Our results showed that neither telomere length nor mtDNA copy number were significantly associated with the symptom severity of SCZ. Further studies are needed to illuminate the roles and mechanisms of telomere and mitochondria in the psychopharmacology and pathogenesis in SCZ.

Several limitations need to be acknowledged. First, 8 weeks of risperidone treatment was relative short to detect the antipsychotic effect on the longitudinal changes of telomere length and mtDNA copy number in SCZ Patients. The limited statistical power may explain the discrepancy between our results and previous studies that suggested risperidone having an adverse effect on mitochondria and a protective effect on telomere^40^,^41^,^42^. Second, we only measured here the telomere length and mtDNA copy number; more studies may help by investigating the association between telomerase activity or mitochondrial electron transport chain (ETC) enzyme activities and the treatment response of SCZ patients.

In conclusion, our study showed that patients with first-episode antipsychotic-naïve SCZ may be associated with mitochondria, but not telomere. Interestingly, our data suggest that telomere length and mtDNA copy number in patients at baseline hold the potential to predict the effectiveness of risperidone treatment. Although the exact pathways of our finding remains unclear and requires further exploration, it may shed light on the understanding of psychopharmacological mechanism of SCZ.

## Methods

### Participants

First-episode antipsychotic-naïve SCZ patients were recruited from the Second Affiliated Hospital of Xinxiang Medical University and the Third People’s Hospital of Yichun. Patients were diagnosed as first-episode SCZ according to the criteria of DSM-IV and were never treated with antipsychotic medications or other psychotropics. The diagnosis of SCZ was further evaluated by a clinical psychiatrist based on the Structured Clinical Interview for DSM- IV Axis I Disorders (SCID-IV). The symptoms of SCZ were assessed by using the Positive and Negative Symptom Scale (PANSS). The exclusion criteria for patients included: any other Axis I Disorders (depression, bipolar disorder, and substance abuse) and any Axis II Disorders; prior antipsychotic treatment; history of serious medical or neurological disorders; family history of mental or neurological disorders; current serious medical disorders, such as cancer, cardiovascular diseases and neurological disorders. Healthy controls were recruited from the local community through advertisement. Current mental status, substance use, history of physical or neurological illness and personal or family history of any mental disorder for all healthy subjects were assessed by unstructured interviews. Subjects with history of mental or neurological disorders, current substance use and serious medical disorders were excluded from the study.

The complete details of the entire study design and procedures were in accordance with the Declaration of Helsinki. Written informed consent was obtained from each participant and consent from each participant’s guardian was also obtained. This study was approved by the ethics committee of the Second Xiangya Hospital of Central South University.

### Medication and clinical assessments

All patients took risperidone monotherapy for 8 weeks. The dose of antipsychotic was decided by the attending psychiatrist according to the clinical status of patients. Compliance with risperidone was monitored by clinical interviews once two week. No serious side effects were reported during the treatment. Psychopathological status of patients was assessed by using the PANSS at baseline and endpoint of 8-week treatment. The percentage of clinical improvement at 8 weeks was calculated using the formula P% = [(P_0_–P_1_)/P_0_] × 100, where P_0_ was PANSS total score at baseline and P_1_ was 8-week follow-up PANSS total score. According to Leucht *et al.* and Obermeier *et al.* PANSS total score were corrected by subtracting 30 points (the minimum value of PANSS scale) to every PANSS ratings before calculating percentage of clinical improvement^43^,^44^. In this study, the treatment response threshold was defined as the percentage of PANSS reduction above 50%.

### Measurement of telomere length and mtDNA copy number

Peripheral blood samples (5 ml) were collected in EDTA tubes and kept at −70 °C before use. Genomic DNA was isolated from 200 μl of each blood sample using the QIAamp DNA Mini Kit (Qiagen, Hilden, Germany) and according to the manufacturer’s protocol. DNA samples were quantified using the Nanodrop 2000 spectrophotometer (Thermo Scientific, Wilmington, DE, USA).

The telomere length was measured by quantitative polymerase chain reaction (qPCR) originally described by Cawthon^45^. Briefly, the qPCR was assayed by using Maxima SYBR Green qPCR Master Mix (Thermo Fisher Scientific, Waltham, MA, USA). The primers for the telomere PCR were 270 nM Tel1 (5′-GGTTTTTGAGGGTGAGGGTGAGGGTGAGGGTGAGGGT-3′) and 900 nM Tel2 (5′-TCCCGACTATCCCTATCCCTATCCCTATCCCTATCCCTA-3′) and for single-copy gene (36B4) PCR were 300 nM 36B4u (5′-CAGCAAGTGGGAAGGTGTAATCC-3′) and 500 nM 36B4d (5′-CCCATTCTATCATCAACGGGTACAA-3′)^45^. The profile of the telomere amplification was 95 °C for 10 min followed by 30 cycles of 95 °C for 15 seconds, 54 °C for 2 minutes. The profile of the 36B4 amplification was 95 °C for 10 min followed by 35 cycles of 95 °C for 15 seconds and 58 °C for 1 minute.

The mtDNA copy number was assayed by using LightCycler® 480 SYBR Green I Master (Roche, Manheim, Germany). The primers for the mtDNA copy number were 200 nM ND1-F (5′-CCCTAAAACCCGCCACATCT-3′) and 200 nM ND1-R (5′-GAGCGATGGTGAGAGCTA- AGGT-3′). The primers for nuclear single-copy gene (HGB) were 167 nM HGB-F (5′-GTGCACCTGACTCCTGAGGAGA-3′) and 167 nM HGB-R (5′-CCTTGATACCAACCTG- CCCAG-3′)^46^. The thermal cycler condition of the ND1 gene amplification was 95 °C for 10 min followed by 30 cycles of 95 °C for 15 seconds, 60 °C for 1 minute. The thermal cycler condition of the HGB gene amplification was 95 °C for 10 min followed by 35 cycles of 95 °C for 15 seconds, 56 °C for 1 minute.

All qPCR were performed on a Roche LightCycler® 480 machine (Roche Applied Science, Mannhein, Germany). Each sample was run in triplicate using 10 ng DNA per 10 μl reaction. The targeted gene (Tel, ND1) and single copy gene (36B4, HGB) PCR reactions were carried out on separate runs with the same samples in the same well positions, and melting curve analysis was performed for every run to verify specificity. The measures of telomere length and mtDNA copy number were determined by the ratio of telomere and mtDNA content to a reference single copy gene copy number (T/S ratio) in each sample relative to a reference sample. The T and S values were determined by the standard curve method using a serially diluted reference DNA (five point) and the T/S ratio was derived from the T and S value for each sample.

### **S**tatistical analysis

All analyses were performed using the Statistical Package for Social Sciences (SPSS, version 20.0 for Windows). Telomere length was natural transformed to adjust for skewed distributions prior to analysis. Demographic characteristics of the SCZ and control groups were compared using chi squared test or Fisher exact test for categorical variables and independent Student’s t-test for continuous variables. Multiple linear regression analysis was applied to analyzed between-group comparisons in telomere length and mtDNA copy number with age and gender as covariates. Repeated measures ANOVAs were performed to test longitudinal within-group changes (Responders and Non-Responders), and group×time (baseline and follow-up) interaction for telomere length and mtDNA copy number. All P-values were two-sided and considered statistically significant at α < 0.05.

## Additional Information

**How to cite this article**: Li, Z. *et al.* Association of telomere length and mitochondrial DNA copy number with risperidone treatment response in first-episode antipsychotic-naïve schizophrenia. *Sci. Rep.* **5**, 18553; doi: 10.1038/srep18553 (2015).

## Figures and Tables

**Table 1:** Demographic and clinical variables for the subjects.

Variables	Schizophrenia (N = 137)	Healthy control (N = 144)	P value
Age (years)	25.46 ± 7.27	26.03 ± 5.19	0.452
Sex (M/F)	95/42	87/57	0.118
Illness of duration (months)	9.80 ± 12.58		
Positive syndromes	24.54 ± 4.80		
Negative syndromes	20.96 ± 6.62		
General syndromes	45.79 ± 8.33		

**Table 2:** Linear regression analyses of natural transformed telomere length and mtDNA copy number in schizophrenia patients and controls.

Parameter	Natural transformed telomere length			MtDNA copy number		
	Beta	SE	P	Beta	SE	P value
Overall						
**Intercept**	0.184	0.080	0.022	1.184	0.101	**<0.001**
**Patient vs control**	0.010	0.027	0.718	−0.108	0.034	**0.002**
**Female vs Male**	0.018	0.028	0.507	0.057	0.035	0.105
**Age**	−0.007	0.002	**0.002**	−0.006	0.003	**0.031**

Bold value indicates significant result (P<0.05).

**Table 3:** Correlation of telomere length and mitochondrial DNA copy number with between symptoms severity at baseline in schizophrenia. All models were adjusted for age, gender, illness duration.

Variables	Baseline natural transformed telomere length		Baseline mtDNA copy number	
	β	P value	β	P value
PANSS subscales				
Positive syndromes	0.411	0.818	−3.389	0.069
Negative syndromes	−0.439	0.855	−1.800	0.477
General syndromes	0.252	0.935	−0.026	0.993
PANSS total syndromes	0.224	0.967	−5.215	0.357

## References

[b1] Epel E. S. *et al.* Cell aging in relation to stress arousal and cardiovascular disease risk factors. Psychoneuroendocrinology 31, 277–287 (2006). 16298085 10.1016/j.psyneuen.2005.08.011

[b2] Farooqui T. & Farooqui A. A. Aging: an important factor for the pathogenesis of neurodegenerative diseases. Mech Ageing Dev 130, 203–215 (2009). 19071157 10.1016/j.mad.2008.11.006

[b3] Kalyani R. R. & Egan J. M. Diabetes and altered glucose metabolism with aging. Endocrinol Metab Clin North Am 42, 333–347 (2013). 23702405 10.1016/j.ecl.2013.02.010 PMC3664017

[b4] Koutsouleris N. *et al.* Accelerated brain aging in schizophrenia and beyond: a neuroanatomical marker of psychiatric disorders. Schizophr Bull 40, 1140–1153 (2014). 24126515 10.1093/schbul/sbt142 PMC4133663

[b5] Verhoeven J. E., Revesz D., Wolkowitz O. M. & Penninx B. W. Cellular aging in depression: Permanent imprint or reversible process?: An overview of the current evidence, mechanistic pathways, and targets for interventions. Bioessays 36, 968–978 (2014). 25143317 10.1002/bies.201400068

[b6] Shay J. W. Telomerase therapeutics: telomeres recognized as a DNA damage signal: commentary re: K. Kraemer *et al.* antisense-mediated hTERT inhibition specifically reduces the growth of human bladder cancer cells. Clin. Cancer Res., 9: 3794-3800, 2003. Clin Cancer Res 9, 3521–3525 (2003). 14506137

[b7] De Lange T. T-loops and the origin of telomeres. Nat Rev Mol Cell Biol 5, 323–329 (2004). 15071557 10.1038/nrm1359

[b8] Mathon N. F. & Lloyd A. C. Cell senescence and cancer. Nat Rev Cancer 1, 203–213 (2001). 11902575 10.1038/35106045

[b9] Green D. R. & Reed J. C. Mitochondria and apoptosis. Science 281, 1309–1312 (1998). 9721092 10.1126/science.281.5381.1309

[b10] Kowaltowski A. J., de Souza-Pinto N. C., Castilho R. F. & Vercesi A. E. Mitochondria and reactive oxygen species. Free Radic Biol Med 47, 333–343 (2009). 19427899 10.1016/j.freeradbiomed.2009.05.004

[b11] Yakes F. M. & Van Houten B. Mitochondrial DNA damage is more extensive and persists longer than nuclear DNA damage in human cells following oxidative stress. Proc Natl Acad Sci USA 94, 514–519 (1997). 9012815 10.1073/pnas.94.2.514 PMC19544

[b12] Carew J. S. & Huang P. Mitochondrial defects in cancer. Mol Cancer 1, 9 (2002). 12513701 10.1186/1476-4598-1-9 PMC149412

[b13] Taylor R. W. & Turnbull D. M. Mitochondrial DNA mutations in human disease. Nat Rev Genet 6, 389–402 (2005). 15861210 10.1038/nrg1606 PMC1762815

[b14] Barazzoni R., Short K. R. & Nair K. S. Effects of aging on mitochondrial DNA copy number and cytochrome c oxidase gene expression in rat skeletal muscle, liver, and heart. J Biol Chem 275, 3343–3347 (2000). 10652323 10.1074/jbc.275.5.3343

[b15] Sahin E. & DePinho R. A. Axis of ageing: telomeres, p53 and mitochondria. Nat Rev Mol Cell Biol 13, 397–404 (2012). 22588366 10.1038/nrm3352 PMC3718675

[b16] Sahin E. *et al.* Telomere dysfunction induces metabolic and mitochondrial compromise. Nature 470, 359–365 (2011). 21307849 10.1038/nature09787 PMC3741661

[b17] Haendeler J. *et al.* Mitochondrial telomerase reverse transcriptase binds to and protects mitochondrial DNA and function from damage. Arterioscler Thromb Vasc Biol 29, 929–935 (2009). 19265030 10.1161/ATVBAHA.109.185546

[b18] Kim J. H., Kim H. K., Ko J. H., Bang H. & Lee D. C. The Relationship between Leukocyte Mitochondrial DNA Copy Number and Telomere Length in Community-Dwelling Elderly Women. PLoS One 8, e67227 (2013). 23785520 10.1371/journal.pone.0067227 PMC3681770

[b19] Tyrka A. R. *et al.* Association of telomere length and mitochondrial DNA copy number in a community sample of healthy adults. Exp Gerontol 66, 17–20 (2015). 25845980 10.1016/j.exger.2015.04.002 PMC4459604

[b20] Kirkpatrick B., Messias E., Harvey P. D., Fernandez-Egea E. & Bowie C. R. Is Schizophrenia a Syndrome of Accelerated Aging? Schizophr Bull 34, 1024–1032 (2008). 18156637 10.1093/schbul/sbm140 PMC2632500

[b21] Kao H. T. *et al.* Rapid telomere erosion in schizophrenia. Mol Psychiatry 13, 118–119 (2008). 18202693 10.1038/sj.mp.4002105

[b22] Mansour H. *et al.* Does telomere length mediate associations between inbreeding and increased risk for bipolar I disorder and schizophrenia? Psychiatry Res 188, 129–132 (2011). 21300409 10.1016/j.psychres.2011.01.010

[b23] Nieratschker V. *et al.* Longer telomere length in patients with schizophrenia. Schizophr Res 149, 116–120 (2013). 23870621 10.1016/j.schres.2013.06.043

[b24] Malaspina D. *et al.* Telomere length, family history, and paternal age in schizophrenia. Mol Genet Genomic Med 2, 326–331 (2014). 25077175 10.1002/mgg3.71 PMC4113273

[b25] Yu W. Y., Chang H. W., Lin C. H. & Cho C. L. Short telomeres in patients with chronic schizophrenia who show a poor response to treatment. J Psychiatry Neurosci 33, 244–247 (2008). 18592039 PMC2441885

[b26] Fernandez-Egea E. *et al.* Telomere Length and Pulse Pressure in Newly Diagnosed, Antipsychotic-Naive Patients With Nonaffective Psychosis. Schizophr Bull 35, 437–442 (2009). 19279086 10.1093/schbul/sbn169 PMC2659310

[b27] Kakiuchi C. *et al.* Quantitative analysis of mitochondrial DNA deletions in the brains of patients with bipolar disorder and schizophrenia. Int J Neuropsychopharmacol 8, 515–522 (2005). 16202181 10.1017/S1461145705005213

[b28] Sabunciyan S. *et al.* Quantification of total mitochondrial DNA and mitochondrial common deletion in the frontal cortex of patients with schizophrenia and bipolar disorder. J Neural Transm 114, 665–674 (2007). 17195919 10.1007/s00702-006-0581-8

[b29] Torrell H. *et al.* Mitochondrial DNA (mtDNA) in brain samples from patients with major psychiatric disorders: gene expression profiles, mtDNA content and presence of the mtDNA common deletion. Am J Med Genet B Neuropsychiatr Genet 162, 213–223 (2013). 23355257 10.1002/ajmg.b.32134

[b30] Kota L. N., Purushottam M., Moily N. S. & Jain S. Shortened telomere in unremitted schizophrenia. Psychiatry Clin Neurosci 69, 292–297 (2015). 25430532 10.1111/pcn.12260

[b31] Martinsson L. *et al.* Long-term lithium treatment in bipolar disorder is associated with longer leukocyte telomeres. Transl Psychiatry 3, e261 (2013). 23695236 10.1038/tp.2013.37 PMC3669924

[b32] Somerville S. M., Lahti A. C., Conley R. R. & Roberts R. C. Mitochondria in the striatum of subjects with schizophrenia: relationship to treatment response. Synapse 65, 215–224 (2011). 20665724 10.1002/syn.20838 PMC4504676

[b33] Somerville S. M., Conley R. R. & Roberts R. C. Mitochondria in the striatum of subjects with schizophrenia. World J Biol Psychiatry 12, 48–56 (2011). 20698738 10.3109/15622975.2010.505662

[b34] Roberts R. C., Barksdale K. A., Roche J. K. & Lahti A. C. Decreased synaptic and mitochondrial density in the postmortem anterior cingulate cortex in schizophrenia. Schizophr Res http://dx.doi.org/10.1016/j.schres.2015.1007.1016 [Epub ahead of print] (2015). 10.1016/j.schres.2015.07.016 PMC4591176 26210550

[b35] Uranova N. *et al.* The ultrastructure of lymphocytes in schizophrenia. World J Biol Psychiatry 8, 30–37 (2007). 17366347 10.1080/15622970600960207

[b36] Vawter M. *et al.* Mitochondrial-related gene expression changes are sensitive to agonal-pH state: implications for brain disorders. Mol Psychiatry 11, 615–679 (2006). 16636682 10.1038/sj.mp.4001830 PMC3098558

[b37] Savolainen K. *et al.* History of mental disorders and leukocyte telomere length in late adulthood: the Helsinki Birth Cohort Study (HBCS). J Psychiatr Res 46, 1346–1353 (2012). 22884422 10.1016/j.jpsychires.2012.07.005

[b38] Epel E. S. *et al.* Accelerated telomere shortening in response to life stress. Proc Natl Acad Sci USA 101, 17312–17315 (2004). 15574496 10.1073/pnas.0407162101 PMC534658

[b39] Furukawa T. A. *et al.* Initial severity of schizophrenia and efficacy of antipsychotics: participant-level meta-analysis of 6 placebo-controlled studies. JAMA Psychiatry 72, 14–21 (2015). 25372935 10.1001/jamapsychiatry.2014.2127

[b40] Maurer I. & Moller H. J. Inhibition of complex I by neuroleptics in normal human brain cortex parallels the extrapyramidal toxicity of neuroleptics. Mol Cell Biochem 174, 255–259 (1997). 9309697

[b41] Balijepalli S., Kenchappa R. S., Boyd M. R. & Ravindranath V. Protein thiol oxidation by haloperidol results in inhibition of mitochondrial complex I in brain regions: comparison with atypical antipsychotics. Neurochem Int 38, 425–435 (2001). 11222923 10.1016/s0197-0186(00)00108-x

[b42] Toriumi K. *et al.* Effect of antipsychotics on telomere length in the hippocampus. Int J Neuropsychopharmacol 17, 103–103 (2014).

[b43] Leucht S., Davis J. M., Engel R. R., Kane J. M. & Wagenpfeil S. Defining ‘response’ in antipsychotic drug trials: recommendations for the use of scale-derived cutoffs. Neuropsychopharmacology 32, 1903–1910 (2007). 17287825 10.1038/sj.npp.1301325

[b44] Obermeier M. *et al.* Should the PANSS be rescaled? Schizophr Bull 36, 455–460 (2010). 19889950 10.1093/schbul/sbp124 PMC2879676

[b45] Cawthon R. M. Telomere measurement by quantitative PCR. Nucleic Acids Res 30, e47 (2002). 12000852 10.1093/nar/30.10.e47 PMC115301

[b46] Xing J. *et al.* Mitochondrial DNA Content: Its Genetic Heritability and Association With Renal Cell Carcinoma. J Natl Cancer Inst 100, 1104–1112 (2008). 18664653 10.1093/jnci/djn213 PMC2720693

